# Evaluation of the Synergistic Potential of Simultaneous Pan- or Isoform-Specific BET and SYK Inhibition in B-Cell Lymphoma: An In Vitro Approach

**DOI:** 10.3390/cancers14194691

**Published:** 2022-09-27

**Authors:** Sina Sender, Ahmad Wael Sultan, Daniel Palmer, Dirk Koczan, Anett Sekora, Julia Beck, Ekkehard Schuetz, Leila Taher, Bertram Brenig, Georg Fuellen, Christian Junghanss, Hugo Murua Escobar

**Affiliations:** 1 Hematology, Oncology, Palliative Medicine, Department of Medicine, Clinic III, Rostock University Medical Center, 18057 Rostock, Germany; 2 Institute for Biostatistics and Informatics in Medicine and Ageing Research, Rostock University Medical Center, 18057 Rostock, Germany; 3 Institute for Immunology, Core Facility Genomics, Rostock University Medical Center, 18057 Rostock, Germany; 4 Chronix Biomedical GmbH, 37079 Goettingen, Germany; 5 Institute of Biomedical Informatics, Graz University of Technology, Stremayrgasse 16/I, 8010 Graz, Austria; 6 Institute of Veterinary Medicine, University of Goettingen, 37077 Goettingen, Germany

**Keywords:** BET, I-BET151, AZD5153, entospletinib, SYK, gene expression, drug combination, lymphoma, DLBCL, BL

## Abstract

**Simple Summary:**

B-cell lymphomas represent the majority of non-Hodgkin lymphomas and are the most common lymphoid malignancies in the Western world. Genetic alterations or epigenetic modulations can lead to tumor initiation and tumor progression. Aside from standard care, targeted, individualized therapies can be highly effective. Here, we evaluated the impact of simultaneous specific inhibition of two key regulators involved in B lymphoid tumor progression. Spleen tyrosine kinase (SYK) is a B-cell receptor-associated kinase acting as a proto-oncogene in B-cell malignancies, while bromodomain and extra-terminal domain (BET) proteins are epigenetic reader proteins involved in histone recognition and transcription regulation. The simultaneous inhibition of SYK and BET showed enhanced anti-proliferative effects, as well as inducing a distinct combination-specific gene expression profile, suggesting SYK and BET inhibition as a promising combination in the treatment of B-cell lymphoma.

**Abstract:**

**Background:** Both bromodomain and extra-terminal domain (BET) proteins and spleen tyrosine kinase (SYK) represent promising targets in diffuse large B-cell (DLBCL) and Burkitt’s lymphoma (BL). We evaluated the anti-lymphoma activity of the isoform-specific bivalent BET inhibitor AZD5153 (AZD) and the pan-BET inhibitor I-BET151 (I-BET) as single agents and in combination with SYK inhibitor Entospletinib (Ento) in vitro. **Methods:** The effect of the single agents on cell proliferation and metabolic activity was evaluated in two DLBCL and two BL cell lines. Proliferation, metabolic activity, apoptosis, cell cycle and morphology were further investigated after a combined treatment of AZD or I-BET and Ento. RNAseq profiling of combined AZD+Ento treatment was performed in SU-DHL-4 cells. **Results:** Both BET inhibitors reduced cell proliferation and metabolic activity in a dose- and time-dependent manner. Combined BET and SYK inhibition enhanced the anti-proliferative effect and induced a G0/G1 cell cycle arrest. SU-DHL-4 demonstrated a pronounced modulation of gene expression by AZD, which was markedly increased by additional SYK inhibition. Functional enrichment analyses identified combination-specific GO terms related to DNA replication and cell division. Genes such as *ADGRA2*, *MYB*, *TNFRSF11A*, *S100A10*, *PLEKHH3*, *DHRS2* and *FOXP1-AS1* were identified as possible key regulators. **Conclusion:** Simultaneous inhibition of BET and SYK enhanced the anti-proliferative effects, and induced a combination-specific gene expression signature.

## 1. Introduction

Targeted therapies by small molecule inhibitors and their combinations have been shown to efficiently affect tumor growth and survival in hematological neoplasias. B-cell receptor-associated kinases (BAKs) like spleen tyrosine kinase (SYK), as well as the bromodomain (BRD)-containing proteins such as the bromodomain and extra-terminal domain (BET) proteins play a critical role in signaling pathway transduction. The specific inhibition of BET and SYK represents a promising tool in hematological neoplasias.

BET co-activators represent a group of epigenetic readers involved in histone recognition and transcription regulation [1,2]. BET proteins consist of a hydrophobic pocket within an acetylated lysine binding site (Ac-K), which can be competitively blocked by BET inhibitors thus preventing their binding to Ac-K chromatin [2,3,4]. Induction of BET inhibitors has been shown to trigger a depletion of BRD4 from promotors and enhancers in DLBCL [5]. Decreased BRD4 has consequently been shown to suppress a number of oncogenes such as c-MYC, BCL-6 and BCL-2 in DLBCL and other hematological malignancies, suggesting BET inhibition may be a valid therapeutic option to consider [5,6,7,8,9].

Early BET inhibitors JQ1 and I-BET762 (GSK525762A) were designed to target the BRD4-NUT (nuclear protein in testis) fusion protein in aggressive NUT midline carcinoma. The specific binding of these inhibitors to BRD4, induces the displacement of BRD4 fusion from chromatin and leads to tumor regression in vivo [10,11]. Following this, the targeting of wild-type BRD4 in hematological malignancies has likewise been shown to result in anti-tumor activity due to proto-oncogene downregulation [6,7,8,9,12].

Based on the promising results of targeting BET proteins in hematological and solid tumors, several BET inhibitors have been developed and are under clinical investigation, although currently without FDA approval (reviewed in [13,14]).

Most prominent BET inhibitors are pan-BET inhibitors, with specificity to more than one representative BET family protein. Representative pan-BET inhibitors are I-BET151 (GSK1210151A) [6], ABBV-075 [15], I-BET-762 (GSK525762) [11] and OTX015 (MK 8628) [16]. The isoform-specific inhibitors in particular, such as the monovalent inhibitors JQ1 [10] and CPI-0610 [17] or the bivalent inhibitors such as AZD5153 or ABBV-744 [18,19], demonstrate specific BET inhibition. To further improve the anti-tumor potential of BET inhibitors, various combinations with immune modulators [20], epigenetic drugs [12,21], DNA damage repair inhibitors [22] or antibodies [23] are under pre-clinical and clinical investigation and have revealed pre-clinical efficacy in lymphoma. In particular, targeting the B-cell receptor (BCR) signaling cascade enables a promising approach for B-cell neoplasias due to their dependency on BCR survival signals [24]. Various small molecules engaging BCR or its downstream signaling, such as BTK [12,23,25,26], CDK [25], PI3K [23,27], BCL-2 [28] or SYK inhibitors [26], have shown synergistic activity with BET inhibition in lymphomas. Thus, combinations with BCR-associated kinases (BAKs) offer high potential for synergistic efficacy. The combination of BET inhibitors with the specific spleen tyrosine kinase (SYK) inhibitor Entospletinib (Ento) appears promising, as SYK is a direct downstream kinase of the BCR and responsible for several cellular processes such as proliferation, differentiation, cytoskeletal remodeling, cytokine release and survival [29].

Here, we aimed to compare the anti-tumorigenic effect of a simultaneous SYK inhibition by Entospletinib with pan-BET inhibition by I-BET151 (I-BET) or isoform-specific bivalent BET inhibition by AZD5153 (AZD) in a DLBCL and Burkitt’s lymphoma in vitro approach. We investigated the synergistic potential of additional specific SYK inhibition and the resulting specific gene expression modulation patterns in both B-lymphoma subtypes. Inhibitor-based transcriptome modulation was evaluated by RNA sequencing and supported by microarray analyses.

We uncovered additional anti-tumorigenic effects in B-lymphoma cell lines induced by simultaneous SYK and BET inhibition, which was investigated by analyzing proliferation, metabolic activity, apoptosis and cell cycle analyses. Cellular processes such as DNA replication and cell cycle were identified as key modulating pathways by high-throughput RNA sequencing.

## 2. Materials and Methods

### 2.1. Human Cell Lines, Reagents and Inhibitor Exposure

Human Burkitt’s lymphoma (BL) cell lines DG-75 and RAJI and diffuse large B-cell lymphoma (DLBCL) cell lines SU-DHL-4 (GCB) and U-2946 (ABC) were purchased from the German Collection of Microorganisms and Cell Cultures (DSMZ, Braunschweig, Germany), cultured as recommended by the manufacturer in RPMI 1640 media (Biochrom, Berlin, Germany) supplemented with 10% heat-inactivated FCS (Biochrom, Berlin, Germany) and 100 µg/mL penicillin and streptomycin (Biochrom, Berlin, Germany). Cultured cells were regularly tested for mycoplasma contamination and authenticity (cell surface markers by flow cytometry).

AZD5153 (AZD), I-BET 151 (GSK1210151A) (I-BET) and Entospletinib (GS-9973) (Ento) were obtained from Selleck Chemicals (Absource Diagnostics GmbH, Munich, Germany) and prepared according to the manufacturer’s instructions to a 10 mM stock and stored at −80 °C until ready for use.

Cell lines (3.33 × 10 ^5^ cells/mL) were exposed to AZD, I-BET, Ento or DMSO (control) as mono substance (0.001 µM–10 µM) or in combination for up to 72 h (0.01 µM AZD, 0.1 µM I-BET, 1 µM Ento)

### 2.2. Cell Proliferation and WST-1 Proliferation Assay

Exposed cells were harvested and washed with PBS (Biochrom, Berlin, Germany). Viable cells were determined by trypan blue staining and cell count with a hemocy-tometer (Sigma-Aldrich Chemie GmbH, Steinheim, Germany). Metabolic activity was determined via tetrazolium compound WST-1 (TaKaRa Bio Inc., Kusatsu, Japan) according to the manufacturer’s protocol and previously described in [30].

### 2.3. Apoptosis Assay

Early and late apoptosis was analyzed by Annexin V FITC (BD Biosciences, Heidelberg, Germany) and Propidium Iodide (PI) (Sigma Aldrich, St. Louis, MO, USA) staining according to the manufacturer’s protocol and previously described in [30]. Measurements were performed using FACS Verse^TM^ (BD Biosciences, Heidelberg, Germany) and BD FACS Suite Software (BD Biosciences, Heidelberg, Germany, Version 4.0.2).

### 2.4. Cell Cycle Analysis

Protocol was previously described in [30]. Briefly, cells were fixed with 70% ice cold ethanol and frozen at −20 °C (≥24 h). Fixed cells were twice washed with PBS and then RNase treated in 500 µL ribonuclease A (1 mg/mL) (Carl Roth, Karlsruhe, Germany) for 45 min at 37 °C. Cells were washed, cell pellet resuspended in 400 µL PI solution (50 µg/mL) (Sigma Aldrich Chemie GmbH, Steinheim, Germany) and analyzed by flow cytometry (FACS Verse^TM^, BD Biosciences, Heidelberg, Germany). Cell cycle distribution was examined by FlowJo Software (Beckton Dickinson, Franklin Lakes, NJ, USA).

### 2.5. May–Grunwald Giemsa Staining

Coated cytoslides (Tharmac, Waldsolms, Germany) were prepared with 5 × 10^4^ cells per slide (700 rpm, 10 min) in a Cytospin 3 centrifuge (Shandon, Frankfurt/Main, Germany). Slides were stained for 6 min with May–Grunwald working solution (Merck, Darmstadt, Germany), rinsed with microscopy buffer according to WEISE (pH 7.2) (Merck, Darmstadt, Germany), stained 20 min in Giemsa working solution (Merck, Darmstadt, Germany) and rinsed thoroughly with microscopy buffer again. The slides were air dried at room temperature before analysis. Morphological changes were analyzed by the EVOS^®^ XL Core Imaging System (AMG, Washington, DC, USA).

### 2.6. RNA Extraction and Isolation

Cell pellets were resuspended in QIAzol Lysis Reagent (QIAGEN, Venlo, The Netherlands) and RNA isolation was performed with miRNeasy Mini Kit (QIAGEN, Venlo, The Netherlands) according to the manufacturer’s instructions and previously described in [30].

### 2.7. RNA Sequencing and Microarray Analyses

Poly-A RNA was enriched from 1 µg total RNA using the NEBNext Poly (A) mRNA Magnetic Isolation Module (New England Biolabs, Ipswich, MA, USA) and sequencing libraries were prepared using the NEBNext Ultra II RNA Library Prep Kit (New England Biolabs, Ipswich, MA, USA). Sequencing was conducted on an Illumina NextSeq500 (Illumina, San Diego, CA, USA) as single reads with 75 bp length with a total of 480 million reads.

Gene expression analyses were also performed by microarray, using the Human Clariom D Array (Thermo Fisher Scientific, Waltham, MA, USA) according to manufacturer’s instructions and previously described in [31]. In brief, RNA quality was assessed on a Bioanalyzer 2100 (Agilent, Santa Clara, CA, USA) and RIN ≥ 9.4 considered as applicable. A total of 200 ng RNA was used for synthesis of first-strand cDNA, followed by second-strand- and cRNA-synthesis. A magnetic bead-based method was used for cRNA purification. Further, an amount of 15 µg cRNA was applied for 2nd-cycle sense strand single-stranded cDNA synthesis, followed by RNase H hydrolyzation, magnetic bead-based purification and quantification. Subsequently, 5.5 µg ss cDNA were fragmented, fragmentation was assessed with a Bioanalyzer 2100 (Agilent, Santa Clara, CA, USA) and end-labeling by biotin attachment was performed. Hybridization was then performed in a GeneChip Hybridization Oven 645 (Affymetrix, Santa Clara, CA, USA) at 45 °C overnight and washed and stained in an Affymetrix Fluidics station 450 (Affymetrix, Santa Clara, CA, USA). Microarrays were scanned in the GeneChip Scanner 3000 7G (Affymetrix, Santa Clara, CA, USA) at 0.7 micron resolution.

### 2.8. RNA Sequencing and Microarray Analysis Pipeline

Microarray data were RMA normalized using limma and probes were mapped to genes using *affycoretools* with probe mappings drawn from *clariomhumantranscriptcluster.db.* and a linear model was fitted of the form:

$$Y_{ij}=\beta_{0j}Treatment_{i}+\beta_{1j}Line_{i}+\beta_{2j}Batch_{i}+\epsilon_{ij}$$

where *Y_ij_* is the intensity for gene *j* in sample I, *Treatment_i_* is the treatment which sample *i* was subjected to, *Line_i_* is the cell line from which sample *i* was drawn, *Batchi* is the batch which sample *i* belongs to and ϵ*_ij_* is the error term, with the coefficients *β*_0_, *β*_1_ and *β*_2_ estimated by least squares. All contrasts were tested using the eBayes method of limma to perform a moderated *t*-test for each gene and the results were corrected for multiplicity using the Benjamini–Hochberg correction.

The RNAseq data were processed as follows: The raw reads from different lanes were concatenated and then trimming and quality control were performed using CutAdapt via TrimGalore! [32]. Trimmed reads were aligned to the human genome (GRCh38.p13) and feature counts were performed using *Rsubread* [33]. *edgeR* was used to filter low read counts using the *filterByExpr()* function, normalized using the TMM method, and a differential expression analysis was performed using the GLM method to fit a generalized linear model, specifying no intercept and with treatment group and batch as covariates. All contrasts were tested using the *glmQLFTest()* function of *edgeR*, which performs an empirical Bayes quasi-likelihood F-test for each gene [34], and the results were corrected for multiplicity using the Benjamini–Hochberg correction. For both the microarray and RNAseq data, a gene was deemed significantly differentially expressed if it had an adjusted *p*-value of <0.05 and a fold-change > 2.

Principal component analysis (PCA) of the microarray data (using the normalized expression values) and the RNAseq data (using logCPM values, calculated setting a prior count of 3) was performed to identify trends in the data. For the PCA plots, the cell batch effect was controlled by first using the removeBatchEffect() function of limma.

Gene ontology (GO) enrichment analysis of biological process terms was performed using Enrichr [35] with a Benjamini–Hochberg adjusted *p*-value of <0.05 being taken as significant. When considering the importance of GO terms, terms were ranked by Enrichr’s combined score. This is calculated by multiplying the log of the *p*-value for a given term by the z-score of the deviation of that term from its expected rank, given the size of the gene set. In this way the combined score aims to better account for biases introduced into enrichment analysis by variation in gene set size.

### 2.9. Platform Comparison

To compare the two platforms, the overlaps between all like gene lists were obtained and can be seen in Supplementary Table S4.

### 2.10. Statistics, Reproducibility and Bliss Calculation

Statistical data are presented as mean  ±  standard deviation (SD) from at least three biologically independent samples. Statistical differences between multiple comparisons were analyzed using one-way ANOVA followed by Tukey’s test as post-hoc analysis for normally-distributed data. The Kruskal–Wallis test was applied to non-parametric data. Differences were considered statistically significant for * *p* < 0.05, ** *p* < 0.01, *** *p* < 0.001.

Synergy was evaluated by a mathematical Bliss independence model [36]. Bliss synergy is defined as EA+EB–EA EB. The difference (Δ) of observed and expected values indicates additive, synergistic or antagonistic effects. Δ = 0, Δ > 0 and Δ < 0 specifies an additive-, synergistic- and antagonistic interaction, respectively.

## 3. Results

### 3.1. Both the Isoform-Specific Bivalent- and Pan-BET Inhibitor Affect Proliferation and Metabolic Activity at Low Dosage in DLBCL and BL Cell Lines

**Figure 1:**
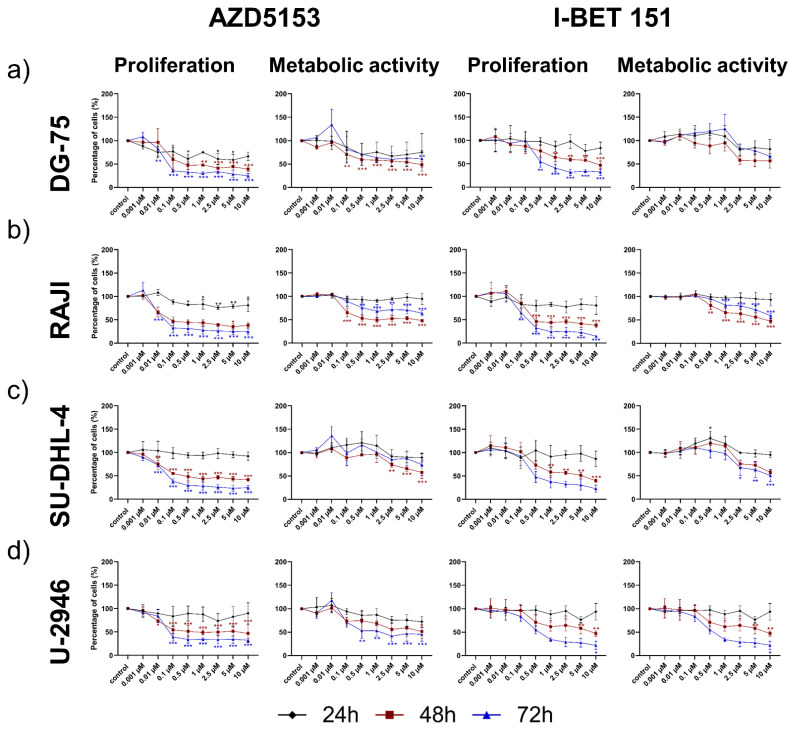
Evaluation of cell viability after isoform-specific- and pan-BET inhibition in Burkitt‘s lymphoma and diffuse large B-cell lymphoma cell lines. Burkitt‘s lymphoma (BL) cell lines (**a**) DG-75 and (**b**) RAJI; and diffuse large B-cell lymphoma (DLBCL) cell lines (**c**) SU-DHL-4 and (**d**) U-2946 were exposed to serial-diluted isoform-specific inhibitor AZD5153 or pan-BET inhibitor I-BET151 (0.001 µM–10 µM). Cell proliferation was determined by trypan blue staining and metabolic activity by WST-1 assay 24 h, 48 h and 72 h after exposure. Data are presented as the mean ± SD. Statistical significance was calculated by one-way ANOVA followed by Dunnett’s multiple comparison test as post hoc analysis. The Kruskal–Wallis test was applied to non-parametric data. Statistical significance is displayed as * *p* < 0.033, ** *p* < 0.002, *** *p* < 0.001 versus control group (n ≥ 3).

Burkitt’s lymphoma (BL) cell lines DG-75 and RAJI and diffuse large B-cell lymphoma (DLBCL) cell lines SU-DHL-4 and U-2946 were exposed to dose ranges of isoform-specific bivalent BET inhibitor AZD5153 (AZD) or pan-BET inhibitor I-BET 151 (I-BET) (0.001–10 µM) for 24 h, 48 h and 72 h (Figure 1). Both inhibitors were able to induce a dose- and time-dependent anti-proliferative effect in all tested cell lines with slightly lower IC50 values for AZD (Supplementary Figure S1). The cell proliferation was most affected after 72 h substance incubation, while metabolic activity showed the strongest reduction after 48 h or 72 h. The respective IC50 values for all tested B-lymphoma cell lines were calculated based on 72 h proliferation and are shown in Supplementary Figure S1.

### 3.2. Entospletinib Reduced Cell Viability of the DLBCL Cell Line SU-DHL-4 Selectively

The DLBCL cell line SU-DHL-4 showed a significant decrease in cell proliferation after 48 h (*p* < 0.05) and 72 h (*p* < 0.01) Entospletinib (Ento) exposure. Metabolic activity was significantly reduced within 48 h but not after 72 h of substance application (Supplementary Figure S2c). IC50 was calculated based on proliferation with 2.67 µM Ento treatment (Supplementary Figure S2e).

In contrast, Ento was not able to reduce cell proliferation and metabolic activity significantly in DG-75, RAJI and U-2946 at the used concentration regimes (Supplementary Figure S2a,b,d, respectively). Accordingly, IC50 values were not reached under the tested conditions for these cell lines.

### 3.3. Simultaneous BET and SYK Inhibition Revealed a Moderate Synergistic Effect Compared to Single Agent Response

Cell viability of combined exposure to Ento and isoform-specific BET inhibitor AZD or pan-BET inhibitor I-BET was carried out after 72 h of incubation with the given substances. The same single agent concentrations were used for the combined exposure for all cell lines for direct comparison (Ento: 1 µM, AZD: 0.01 µM and I-BET: 0.1 µM).

**Figure 2:**
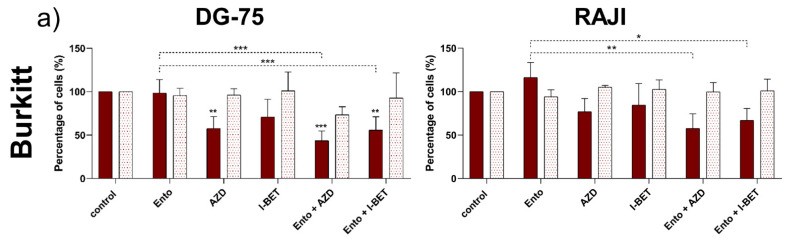

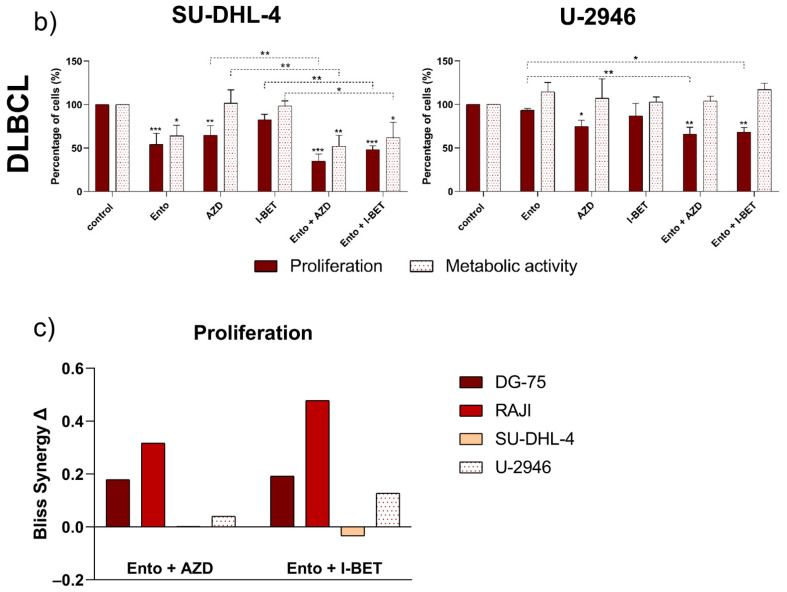
Simultaneous BET and SYK inhibition affected cell viability of BL and DLBCL cell lines synergistically. Cells were exposed to 1 µM Ento, 0.01 µM AZD or 0.1 µM I-BET as a single agent or in combination. Cell proliferation and metabolic activity were assessed in (**a**) BL cell lines (DG-75 and RAJI) and (**b**) DLBCL cell lines (SU-DHL-4 and U-2946) after 72 h. (**c**) Bliss calculations were based on proliferation. Synergistic effects of both combinations were calculated with a Bliss Independence model (EA+EB–EAEB). A value Δ = 0, >0 and <0 defines an additive-, synergistic- or antagonistic interaction, respectively. Data are presented as the mean ± SD. Statistical significance of cell viability data was calculated by one-way ANOVA followed by Tukey’s multiple comparison test as a post hoc analysis and displayed as * *p* < 0.033, ** *p* < 0.002, *** *p* < 0.001 versus control group and further versus the respective single agents (dashed line) (n ≥ 3).

Both combinations (Ento+AZD; Ento+I-BET) induced a significant reduction of cell proliferation after 72 h exposure compared to the DMSO control in DG-75, SU-DHL-4 and U-2946 (Figure 2a,b). Cell proliferation of RAJI was slightly more affected by the combinations, but without a significant decrease compared to the single agents.

However, compared to the Ento single agent, the combinations induced an additional anti-proliferative effect in DG-75, RAJI and U-2946. Further, in SU-DHL-4 the combination induced a significant reduction of proliferation and metabolic activity compared to both BET inhibitors as single agents.

Furthermore, both combinations reduced metabolic activity significantly in the DLBCL cell line SU-DHL-4 compared to the DMSO control and AZD or I-BET single agents, respectively (Figure 2b). However, metabolic activity of DG-75, RAJI and U-2946 was not additionally affected by the combined exposures (Figure 2a,b).

The synergistic potential of BET and SYK inhibition in BL and DLBCL cell lines was evaluated by a Bliss independence model (Figure 2c based on 72 h proliferation). Both combinations (AZD+Ento and I-BET+Ento) revealed positive Bliss values for the BL cell lines DG-75 and RAJI as well as the DLBCL cell line U-2946 indicating a slight synergistic effect. For SU-DHL-4 an additive effect was observed for AZD+Ento with a Bliss value of 0.002. The I-BET+Ento combination showed a minor antagonistic effect with a Bliss value of −0.03. Bliss calculations based on cell cycle distribution are represented in Supplementary Figure S6.

### 3.4. Simultaneous BET and SYK Inhibition Modulates Cell Morphology Moderately

**Figure 3:**
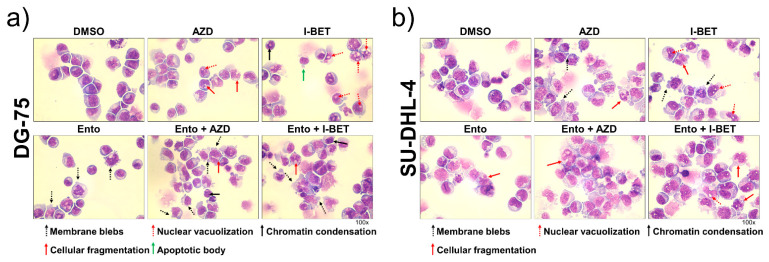
Morphological characterization after BET and SYK inhibition. Cells were exposed to 1 µM Ento, 0.01 µM AZD or 0.1 µM I-BET as a single agent or in combination. Representative light microscopy images of exposed cells after 72 h incubation. Cytospins were stained with May–Grunwald Giemsa stain (Pappenheim method). Light microscopy images (×100) revealed only moderate morphological changes in (**a**) BL cell line DG-75 and (**b**) DLBCL cell line SU-DHL-4.

Cell morphology of the BL cell line DG-75 and the DLBCL cell line SU-DHL-4 was investigated by May–Grunwald Giemsa (Pappenheim) staining at 72 h (Figure 3a,b). The enlarged figures can be found in the supplements (Supplementary Figures S3 and S4). In DG-75 AZD and I-BET single agent exposure provides marginal indications for apoptosis and stress induction such as nuclear vacuolization, chromatin condensation and cellular fragmentation. Ento, in contrast, particularly induced membrane blebs. The combined exposure only moderately intensified morphological changes. Likewise, the SU-DHL-4 cell line provided evidence for slight apoptosis and stress induction after treatment, with particular changes of nuclear vacuolization and cellular fragmentation.

### 3.5. Simultaneous BET and SYK Inhibition-Induced Cell Cycle Blockade in DLBCL and BL Cell Lines, but Not Apoptosis

Moreover, we investigated if simultaneous inhibition of BET and SYK is able to boost the effect on cell cycle blockade after 72 h exposure, compared to the single agents.

**Figure 4:**
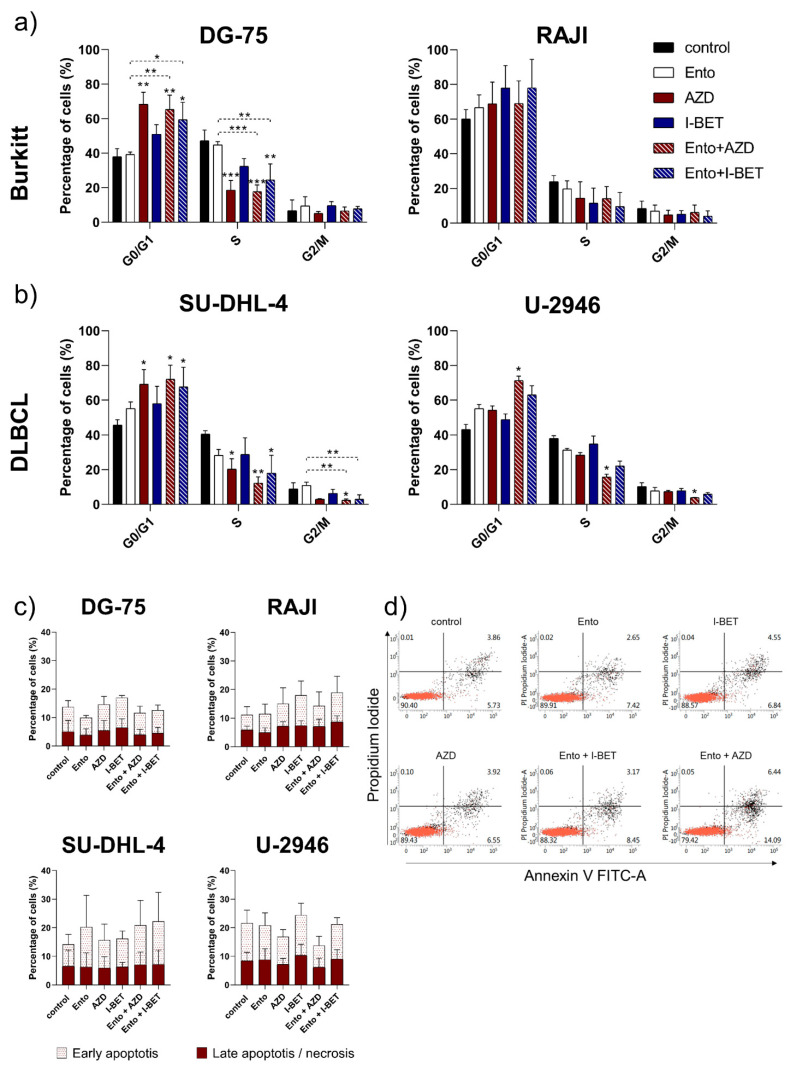
Cell cycle analysis and apoptosis measurement after combined BET and SYK inhibition. Cell cycle analysis was performed 72 h after agent exposure by PI staining and DNA content was measured by flow cytometry. Percentage of cells per cell cycle phase was calculated using FlowJo Software. (**a**) Mean cell cycle distribution of BL cell lines. (**b**) DLBCL cell lines. (**c**) Apoptosis was measured by Annexin V/PI double staining 72 h after incubation. (**d**) Representative dot plots of apoptosis induction in the SU-DHL-4 cell line. Data are presented as the mean ± SD. Statistical significance was calculated by one-way ANOVA followed by Tukey’s multiple comparison test as a post hoc analysis. Kruskal–Wallis test was applied to non-parametric data. Statistical significance is displayed as * *p* < 0.033, ** *p* < 0.002, *** *p* < 0.001 versus control and further versus the respective single agents (dashed lines) (n ≥ 3).

AZD as a mono application significantly increased the percentage of cells in G0/G1 phase in the BL cell line DG-75 and the DLBCL cell line SU-DHL-4 (Figure 4a,b). The AZD+Ento combination enhanced this effect selectively in U-2946 leading to a G0/G1 cell cycle blockade compared to control (Figure 4b).

Application of I-BET as a single agent did not induce cell cycle changes, while the I-BET+Ento combination significantly intensified the G0/G1 blockade compared to control as well as to the single agents in DG-75, SU-DHL-4.

However, the cell cycle of BL cell line RAJI was not significantly affected (Figure 4a). Representative figures of cell cycle distribution are shown in Supplementary Figure S5a. The sub-G1 cell population only increased significantly in SU-DHL-4 after Ento+AZD exposure and in AZD-treated RAJI cells (Supplementary Figure S5b).

To evaluate if Ento, AZD, I-BET as single agent or in combination induce apoptosis in BL or DLBCL cell lines after 72 h exposure, Annexin V/PI double staining was performed and analyzed by flow cytometry. During the tested conditions, no significant apoptosis induction could be observed for any tested lymphoma cell lines after 72 h (Figure 4c,d).

### 3.6. Combined BET and SYK Inhibition Boost the Changes in Gene Expression

**Figure 5:**
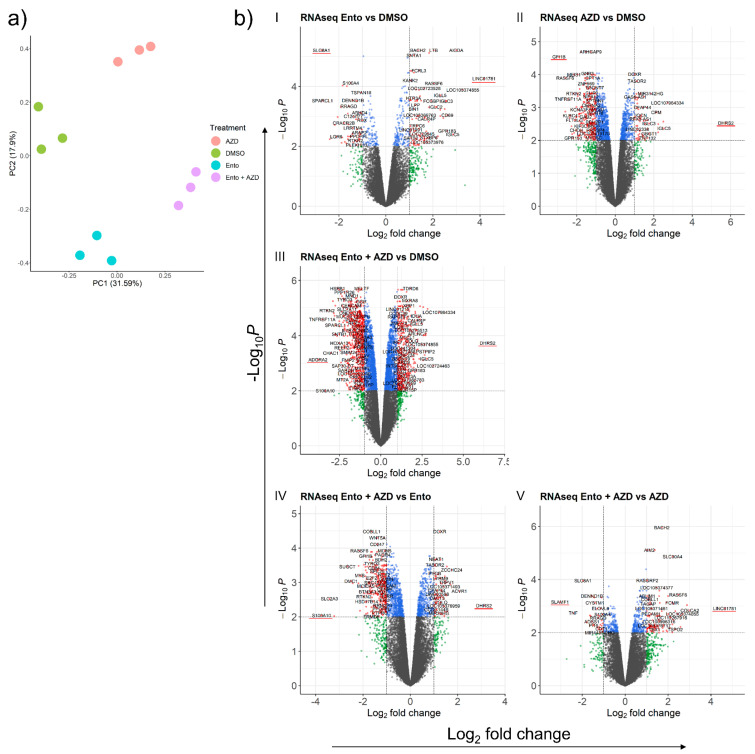
Gene expression modulation in SU-DHL-4 identified by RNAseq. Intensification of gene expression modulation by combined Ento+AZD exposure (**a**) PCA plot of the normalized RNAseq data (logCPM), showing the distinct clustering of the exposure groups in SU-DHL-4 cells (**b**) Volcano plots of the differential expression analysis of the RNAseq dataset for (**I**) Ento vs. DMSO, (**II**) AZD vs. DMSO, (**III**) Ento+AZD vs. DMSO, (**IV**) Ento+AZD vs. Ento and (**V**) Ento+AZD vs. AZD. Highest ranked DEG (overexpressed and underexpressed) is underlined (red) (n = 3).

RNA sequencing was performed in order to evaluate the underlying molecular mechanism of simultaneous BET and SYK inhibition in the DLBCL cell line SU-DHL-4. Principal component analysis (PCA) of exposed SU-DHL-4 cells (after batch correction) showed a distinct clustering of the treatment groups (Figure 5a).

Volcano plots of the differential gene expression analysis highlight the significant deregulated genes by each respective treatment. In particular, Ento+AZD combined exposure (Figure 5b(III)) demonstrated a distinct increase of significantly deregulated genes and a slightly enhanced logFc range compared to the DMSO control. The simultaneous Ento+AZD exposure induced 1285 significantly differentially expressed genes (DEGs) in total (765 down- and 520 upregulated genes with a fold change range of −3.80 to 6.47 (Supplementary Table S1e,f).

Ento and AZD single agent exposures vs. DMSO control (Figure 5b(I,II)) modulated a lower number of genes. Ento exposure induced a significant deregulation of 251 genes in total (83 downregulated and 168 upregulated genes). The observed expression fold change ranged between −2.73 and 4.17 after Ento exposure (Supplementary Table S1a,b). The comparison of AZD vs. DMSO revealed 480 significantly deregulated genes (374 downregulated and 106 upregulated genes) with a fold change range of −2.92 to 5.75 (Supplementary Table S1c,d).

Furthermore, comparing Ento+AZD combined exposure to the single agents Ento (Figure 5b(IV)) or AZD (Figure 5b(V)) demonstrated a specific gene deregulation, representing the significantly and exclusively deregulated genes arising by the respective single agent. The condition Ento+AZD vs. AZD (68 downregulated and 190 upregulated genes) represents fewer deregulated genes compared to Ento+AZD vs. Ento (384 downregulated and 132 upregulated genes), indicating that most modulation was induced by AZD (Supplementary Table S1g–j).

### 3.7. RNAseq Data Validation by Microarray Analyses—Platform Comparison

To validate the RNAseq data, Human Clariom D Array analyses (Thermo Fisher Scientific, Waltham, MA, USA) were carried out for the DLBCL cell line SU-DHL-4 and the BL cell line DG-75 (Supplementary Figures S7–S11 and Tables S2–S4) affirming the RNAseq data, indicating similar findings, while RNAseq seemed to be the more sensitive method. Direct platform comparison was carried out by looking at the top 10 DEGs based on microarray and exposed SU-DHL-4 cells (Supplementary Table S4).

Within the Ento+AZD combination, we found the genes of the long non-coding RNA (lncRNA) FOXP1 antisense RNA 1(*FOXP1-AS1*) and solute carrier family 30 member 4 (*SLC30A4*) highly overexpressed by both procedures.

Furthermore, MYB proto-oncogene, transcription factor *MYB,* leucine rich repeat containing 2 (*LRRCC*), E2F transcription factor 8 (*E2F8*) and chromatin assembly factor 1 subunit B (*CHAF1B*) were identified as highly downregulated by both tools and in both cell lines.

Ento single agent exposure revealed activation-induced cytidine deaminase (*AICDA*) and BTB domain and CNC homolog 2 (*BACH2)* genes as significantly overexpressed, affirmed by both the RNAseq and microarray analyses. In DG-75, *AICDA* was also significantly upregulated by Ento single agent exposure. However, SPARC-like 1 (*SPARCL1*) was identified as significantly downregulated in SU-DHL-4.

AZD single agent only showed overlaps in downregulated genes. Here, Cordon-Bleu WH2 repeat protein-like 1 (*COBLL1*), Ras-association domain family member 6 (*RASSF6*) and Rhotekin 2 (*RTKN2*) were identified as significantly downregulated, while *COBLL1* and *RTKN2* were also found to be significantly downregulated in DG-75.

Throughout, the RNAseq analyses revealed a higher number of DEGs, indicating RNAseq as the more sensitive method. Thus, all following analyses were based on the RNAseq data analyses.

### 3.8. Ento+AZD Combination Intensified the Gene Expression Changes Compared to the Single Agents and Induced a Specific Gene Set Modulation

**Figure 6:**
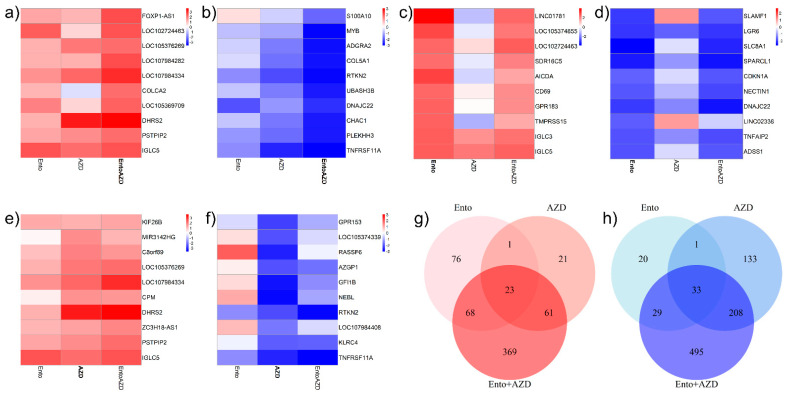
Top 10 over- and underexpressed DEGs and gene intersections in SU-DHL-4 by RNAseq. Heatmaps indicating differential expression of the top 10 DEGs by the respective condition. (**a**) Top 10 overexpressed genes induced by Ento+AZD combined exposure compared to the both single agents in SU-DHL-4 and (**b**) Top 10 underexpressed genes, respectively. (**c**) Top 10 overexpressed genes induced by Ento single agent compared to AZD single agent and Ento+AZD combination and (**d**) Top 10 underexpressed genes, respectively. (**e**) Top 10 overexpressed genes induced by AZD single agent compared to Ento single agent and Ento+AZD combination and (**f**) Top 10 underexpressed genes, respectively. Color range indicates respective |logFC| values. Bold print condition indicates the condition which is analyzed. Treatment conditions in SU-DHL-4 revealed gene intersections and uniquely deregulated genes. (**g**) Gene intersections of overexpressed genes between the treatment conditions (Ento single agent, AZD single agent, Ento+AZD combination) in SU-DHL-4 cells (**h**) Gene intersections of underexpressed genes between the treatment conditions (Ento single agent, AZD single agent, Ento+AZD combination) in SU-DHL-4 cells.

The Ento+AZD combination induced a distinct enhanced gene modulation compared to both the single agents, indicated by a greater number of DEGs as well as increased logFC values. The top 10 genes significantly modulated by the Ento+AZD combination are highlighted in Figure 6a,b compared to the differential gene expression of the single agents (all significant DEGs are listed in Supplementary Table S1e,f). Figure 6c–f illustrates the top 10 DEGs of the single agents Ento and AZD including the compared differential expression of the other single agent and the combined exposure (all significant DEGs are listed in Supplementary Table S1a–d).

The gene intersections of the treatment conditions revealed overlapping and uniquely significantly differentially expressed genes (Figure 6g,h). The Ento+AZD combination demonstrated the most exclusively differentially expressed genes for both the over- and underexpressed directions (Figure 6g,h and Supplementary Table S5). The largest overlaps (208 DEGs) were identified in the underexpressed direction between single agent AZD single agent and Ento+AZD combination (Figure 6h and Supplementary Table S5c).

However, the Ento+AZD combination revealed a specific gene set modulation, since the majority of differentially expressed genes for both under- and overexpressed genes (369 and 495 genes, respectively) were unique to this treatment.

### 3.9. Gene Ontology Enrichment Analyses Identified DNA Replication as the Biological Process Most Modulated by the Ento+AZD Combined Exposure

Gene ontology (GO) enrichment analyses by Enrichr identified several GO terms for the respective condition in the DLBCL cell line SU-DHL-4.

**Figure 7:**
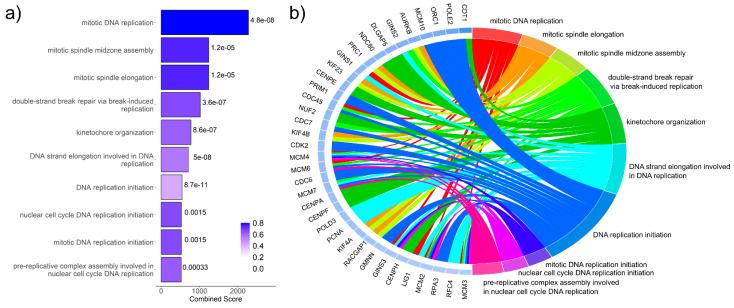
Significant gene ontology term enrichment in Ento+AZD exposed SU-DHL-4 cells by RNAseq. Significant gene ontology (GO) enrichment analyses by Enrichr in Ento+AZD exposed SU-DHL-4 cells. (**a**) Top 10 significantly enriched GO terms of underexpressed genes after Ento+AZD exposure. GO terms were ranked by the combined score. The color gradient indicates the decreasing fraction of significant genes, while darkest color represents the highest significant fraction of genes. <0.05 = significant. n.s. = not significant. (**b**) Chordplot of top 10 enriched GO terms of underexpressed genes. GO terms were ranked by combined score and top 10 significantly downregulated GO terms are illustrated and linked to the significant DEGs. The genes are ranked by logFC, with the relative strength of the logFC indicated by the color adjacent to the gene name (blue indicates underexpression, and a darker color indicates a stronger logFC).

The combination of Ento and AZD induced a significant downregulation of several genes, allocated to GO terms, which are mostly related to DNA replication and cell cycle processes (Figure 7a and Supplementary Table S6e). However, the combination induced no significant enrichment of overexpressed genes (Supplementary Table S6f and Figure S13).

Single agent exposures revealed no significantly enriched GO terms at the adjusted *p* < 0.05 level. The Ento+AZD combination revealed no enriched terms for the overexpressed differentially expressed genes but a total of 120 enriched terms for the underexpressed genes (Supplementary Table S6b,d,f).

A further approach was to identify which of the most strongly downregulated DEGs were allocated to the top 10 significant GO terms (Figure 7b and Supplementary Figure S14). This analysis identified which strongly downregulated DEGs contributed to the enrichment of the top GO terms, helping to visualize and clarify the most significant transcriptomic changes. All of the top allocated genes have pivotal roles in DNA replication processes. Furthermore, different gene sets were identified such as mini chromosome maintenance (*MCM*), centromer (*CEN*), cell division cycle (*CDC*), kinesin family (*KIF*), GINS complex subunit (*GINS*), having more than one gene allocated to the top 10 GO terms. Furthermore, GO term intersections between the conditions are summarized in Supplementary Figure S15 and Tables S7–S9.

### 3.10. GO Term Clusters Revealed DNA Replication and Cell Cycle as the Most Affected Cell Biological Processes by the Ento+AZD Combination

**Figure 8:**
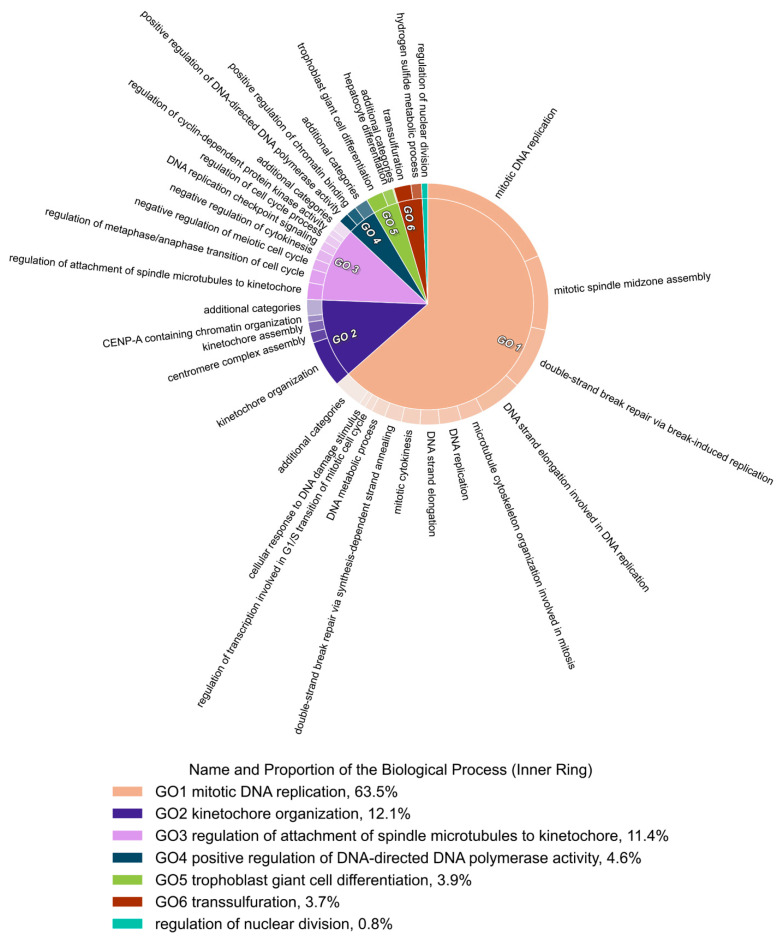
Identified gene ontology clusters after Ento+AZD combined exposure. Gene ontology terms were clustered by CirGO tool. Legend indicates the representative GO terms (GO clusters). Underexpressed GO term cluster after Ento+AZD combined exposure.

Furthermore, GO terms were condensed using REVIGO and subsequently visualized by CirGO identifying GO clusters. Figure 8 highlights the most significant GO term clusters for the Ento+AZD underexpressed condition which are allocated into six representative GO terms (GO clusters) and one non-clustering GO term.

Three big clusters were identified revealing DNA replication, kinetochore organization and cell cycle as the most affected biological processes (Figure 8). Most GO terms (>50%) were associated with representative GO cluster 1 “mitotic DNA replication”. This GO cluster mainly contains GO terms related to DNA replication, double-strand break repair and cell cycle.

## 4. Discussion

Epigenetic changes and deregulated BCR signaling by molecular lesions are able to promote B-lymphomagenesis [37,38,39]. The target-specific inhibition of epigenetic readers like BET or B-cell receptor-associated kinases (BAKs) such as the spleen tyrosine kinase (SYK) enables interference of the associated pathway signal transduction.

Here, we comparatively evaluated the anti-tumorigenic effect of isoform-specific bivalent BET inhibitor AZD5153 and pan-BET inhibitor I-BET151 in a DLBCL and Burkitt’s lymphoma in vitro approach and further investigated the cell- and molecular effect of simultaneous SYK inhibition by Entospletinib.

### 4.1. Both BET Inhibitors Efficiently Affected Cell Proliferation in B-Lymphoma Cell Lines

Both, AZD5153 and I-BET151 as single agents efficiently reduced proliferation and metabolic activity at low dosage in all tested BL and DLBCL cell lines, with slightly lower IC50 values for AZD compared to I-BET. Compared to Rhyasen et al., our evaluated IC50 values were slightly higher for AZD [18]. Further, we have recently shown that the comparative approach of AZD and I-BET in a canine DLBCL in vitro model also revealed stronger anti-proliferative effects by specific bivalent BET inhibition by AZD exposure [40]. This effect was already suggested by simultaneous ligation of both BRD4 bromodomains and the associated efficient and prolonged displacement of BRD4 from chromatins [18,41].

### 4.2. Simultaneous BET and SYK Inhibition Additionally Affected Cell’s Response

Following on from this, the simultaneous exposure of BET inhibitor AZD or I-BET with SYK inhibitor Entospletinib to the BL and DLBCL cell lines further affects proliferation. A pronounced effect was observed in DLBCL cell line SU-DHL-4 by Ento addition, revealing additionally reduced proliferation and metabolic activity. However, the corresponding Bliss values of both combinations showed no synergistic effect, due to the good response to Ento single agent exposure by SU-DHL-4 cells. Ento addition in all other tested cell lines improved anti-proliferative activity moderately, while respective positive Bliss values indicate slight synergy due to the low response to Ento single agent exposure. In a recent study by Kim et al., it was shown that BET inhibition by GS-5829 in combination with Ibrutinib (BTK inhibitor) or Entospletinib synergistically increased anti-leukemia activity and induced apoptosis in primary co-cultured CLL cells [26]. Combinations with several PI3K-pathway-specific inhibitors also revealed additional anti-proliferative activity in a panel of B-lymphoma cell lines in vitro due to the upregulation of PI3K pathway components following BET inhibition [42].

Moreover, we identified a significant G0/G1 cell cycle arrest induced by both combinations in BL cell lines DG-75 and DLBCL cell line SU-DHL-4 compared to control. In DLBCL U-2946 only Ento+AZD induced a significant cell cycle arrest. However, the comparison of both combinations to the single agents only slightly increased the cell cycle blockade.

Similar findings were observed for BET inhibition as a single agent with BRD4 inhibitor JQ1, inducing a G1 cell cycle arrest in DLBCL cell lines [5]. JQ1 was also able to increase the percentage of cells in G1 in RAJI cells, which is in contrast to our findings [8]. Comparing our findings to Takimoto-Shimomura et al., BET inhibition by AZD-induced a G1/S cell cycle blockade in a double hit lymphoma-derived cell line, STR-428, without significantly affecting apoptosis induction [41]. SYK inhibition by PRT060318 on sensitive DLBCL cell lines further suggested affecting the cell cycle but not apoptosis induction [43]. This agrees with our results, indicating that Ento and BET inhibition as single agents did not induce significant apoptosis, while the combined exposure likewise was not able to enhance this effect under the tested conditions.

### 4.3. Simultaneous BET and SYK Inhibition Identified a Combination-Specific Gene Signature in DLBCL Cell Line SU-DHL-4

In order to clarify the underlying molecular mechanism, we further investigated the gene expression modulation and functional enrichment of single agents Ento and AZD and the specific gene set modulation by the Ento+AZD combination.

By RNAseq analyses we identified that both single agents induced gene expression changes, while the combination was capable of highly increasing the gene expression modulation compared to control, as well as to both single agents. The fold change range of the combination likewise increased compared to the single agents. Rhyasen et al. investigated the effect of AZD5153 in xenograft leukemia and lymphoma models and also performed RNAseq on a hematologic cell line panel revealing 174 differentially expressed genes (DEGs) across this panel [18]. Compared to our study, Rhyasen et al. found lower numbers of DEGs presumably due to other treatment conditions and a different analysis approach.

Within the Ento+AZD combination, we identified the adhesion G protein-coupled receptor A2 (*ADGRA2*) as the most significantly underexpressed gene in SU-DHL-4 cells. *ADGRA2* or TEM5/GPR124 (tumor endothelial marker 5) was identified as an orphan G protein-coupled receptor in endothelial cells with roles in brain angiogenesis and Wnt signaling [44,45]. In pancreatic cancer it has been shown that patients with alterations in *ADGRA2* had a worse overall survival [46]. High expression of TEM5 in colorectal cancer has likewise been related to poor survival as well as suggested for use as a progression- and bio-marker [47]. Furthermore, the altered expression of *ADGRA2* in glioblastoma significantly decreased the proliferation of the cancer cells by disrupting the mitotic progression and chromosome segregation [48], while TEM5 also plays a role in VEGF-induced tumor angiogenesis [49]. Indeed, high expression of *ADGRA2* and its role in lymphoma is unknown. However, the DLBCL cell line SU-DHL-4 exhibits an *ADGRA2* missense mutation inducing a protein change p.A765T [50]. According to the Cosmic database, the protein change is unknown. Whether or not this mutation influences the *ADGRA2* expression remains to be clarified.

A further significant downregulation has been observed in the gene TNF receptor superfamily member 11a (*TNFRSF11A*). This receptor regulates various biological processes such as apoptosis, cell survival and differentiation through the activation of several signal transduction pathways such as nuclear factor κB (NF-κB), Jun N-terminal kinase (JNK), p38, extracellular signal-related kinase (ERK) and phosphoinositide 3-kinase (PI3K) [51,52]. Aberrant *TNFRSF11A* (RANK) expression has been linked to several cancers. Its overexpression is correlated to breast cancer and prostate cancer with high metastatic potential and to gliomas [53,54,55]. It further correlates with metastasis and poor prognosis in colon cancer and can act as a prognostic factor [56,57,58]. Together with the stem cell marker KLF5, *TNFRSF11A* induces cancer cell proliferation, migration and invasiveness in cervical cancer [55]. However, *KLF5* was not significantly affected by any of the treatments. Finally, *TNFRSF11A* induces survival and proliferation pathways in B cells in vitro with progression to B-cell malignancies [59]. The significant downregulation of *TNFRSF11A* could be one reason for the effective anti-proliferative effect by the single agents and the intensification by Ento+AZD combination in SU-DHL-4 cells.

The Ento+AZD combination was exclusively able to significantly downregulate the S100 calcium binding protein A10 (*S100A10*). *S100A10* is involved in various cellular processes, regulates plasminogen activation [60] and has been shown to suppress pro-apoptotic capacity of Bcl-2-associated death protein (BAD), which is suggested to have anti-apoptotic function in cancer cells (reviewed in [61]). Furthermore, *S100A10* expression is correlated with tumor development, invasion, poor prognosis and further can act as a biomarker due to its expression in various tumor cells [61,62,63,64].

The combined exposure additionally revealed *MYB* (MYB proto-oncogene, transcription factor) as highly underexpressed. The transcription regulator is known to be aberrantly expressed or rearranged in leukemias, lymphomas and also solid tumors [65,66]. SU-DHL-4 exhibits aberrant overexpression of *MYB* [67], which was decreased by the Ento+AZD combined exposure. It has already been shown that *MYB* downregulation inhibits cell proliferation and invasion in solid tumors [68,69].

Pleckstrin homology, MyTH4 and FERM domain containing H3 (*PLEKHH3)* were also identified as significantly downregulated. The function of *PLEKHH3* is not yet understood, while pleckstrin homology (PH) domains are known to be part of several proteins, e.g., cytoskeletal proteins, function in signal transduction, mediating protein–protein interactions and membrane association [70,71]. Overexpression of *PLEKHH3* was identified in blood immune cells associated with obstructive sleep apnea [72], while the role of *PLEKHH3* in cancer has not been described until now.

However, within the Ento+AZD combination we identified that the gene dehydrogenase/reductase 2 (*DHRS2*) was highly and significantly upregulated. High *DHRS2* expression was observed in BET inhibitor resistant liver cancer cell lines. The specific BET inhibition in liver cancer cells induced an increase of *DHRS2* expression in sensitive (Hep3B) and resistant cells (HepG2). This could be assumed to be related to drug escape mechanisms [73]. Other studies suggest a tumor suppressor function [74,75]. The downregulation of *DHRS2* was further related to invasion, lymph node metastasis and is associated with worse prognosis in esophageal squamous cell carcinoma. The overexpression of *DHRS2* leads to decreased tumor proliferation and tumor volume both in vitro and in vivo, while the *DHRS2* knockdown increases it [75]. In ovarian cancer, HDAC inhibition likewise increased *DHRS2* levels, which was linked to HDACi sensitivity [76]. However, the function of *DHRS2* needs to be clarified in DLBCL.

Furthermore, the colorectal cancer-associated 2 gene (*COLCA2*) was significantly upregulated after combined Ento+AZD exposure in SU-DHL-4 cells. Currently, detailed knowledge about the actual function of *COLCA2* is limited. However, it is assumed to have tumor suppressor function and low expression has been correlated to colorectal cancer [77,78].

The function of both genes *DHRS2* and *COLCA2* in B-cell lymphoma is unknown. Due to its proposed function as a tumor suppressor, the high expression suggests a possible slowing effect on tumor progression.

### 4.4. Overlapping DEGs between RNAseq and Microarray Platform in DLBCL Cell Line SU-DHL-4

To affirm the RNAseq data, we further analyzed the overlapping DEGs of RNAseq and microarray analyses. Genes identified by both analyses are highly likely to play pivotal roles in Entospletinib and AZD5153 exposure.

For example, *MYB* downregulation and *SLC30A4* upregulation by Ento+AZD, *AICDA* upregulation by Ento single agent and *COBLL1* and *RTKN2* downregulation by AZD single agents can be supposed to be part of a general molecular mechanism-induced by the respective substance, emerging in DLBCL SU-DHL-4 as well as BL cell line DG-75.

Compared to the RNAseq analyses, the number of identified deregulated genes are distinctly lower in SU-DHL-4 and DG-75 by microarray analyses. However, Ento exposure in DG-75 only identified *AICDA* as significantly upregulated. This small effect on gene modulation is in concordance with the investigated biological parameters in DG-75 after exposure, where Ento was not able to induce a significant anti-proliferative effect. It can be assumed that Ento exposure as well as Ento+AZD combination is very effective in gene modulation in DLBCL cell lines but less so in BL cell lines.

### 4.5. Comparison of Identified DEGs Affected by Ento+AZD Combinaton with External Gene Lists

Additional validation of the identified DEGs was carried out by comparing to external canine and human data (Supplementary Figure S12). Potentially important DEGs were compared to published results from two different canine primary B-cell lymphoma sample cohorts [79,80].

Canine primary B-cell lymphoma samples showed underexpression of *ADGRA2* compared to non-neoplastic controls [79]. In glioblastoma the alteration of *ADGRA2* expression reduced cell proliferation [48]. It could be suggested, that the alteration of *ADGRA2* expression in DLBCL cell line SU-DHL-4 likewise induced anti-proliferative effects.

Aberrant *MYB* expression has been identified in several tumors and leukemias [65,66]. DLBCL cell line SU-DHL-4 also showed high *MYB* expression [67]. Likewise, *MYB* showed high expression in canine primary B-cell lymphoma samples [79]. As *MYB* downregulation inhibits cell proliferation and invasion in solid tumors [68,69], it could be also suggested to influence the anti-proliferative effects in SU-DHL-4.

*TNFRSF11A* overexpression is linked to several cancers [53,54,55]. *TNFRSF11A* expression in the canine primary B-cell lymphoma samples was also shown to be underexpressed compared to non-neoplastic primary material [79].

*AICDA* is underexpressed in the canine primary B-cell lymphoma samples compared to the non-neoplastic primary material, except for two outliers with high *AICDA* expression [79]. The study by Mudaliar et al. likewise indicates *AICDA* underexpression in canine DLBCL samples [80]. In our study, *AICDA* is upregulated by the Ento single agent exposure and slightly upregulated by the combination of Ento and AZD. Due to the fact that *AICDA* expression has been correlated to poor outcome in DLBCL patients [81], it could indicate a drug escape mechanism induced by the Ento single exposure.

### 4.6. Gene Ontology (GO) Enrichment Analyses Identified Combination-Specific Biological Processes

Gene ontology (GO) enrichment analyses identified the main biological processes which were affected after combined or single agent exposure in DLBCL cell line SU-DHL-4. For the Ento+AZD combination, GO term enrichment analysis identified combination-specific GO terms compared to the single agents. Furthermore, only the underexpressed results of Ento+AZD combined exposure revealed several highly significantly modified GO terms. All other conditions showed no significance, but sometimes a high combined score.

The identified downregulation of general biological processes is related to DNA replication and cell division processes which may be caused by cell death induction due to combined BET and SYK inhibition. This assumption can be affirmed by the observed significant downregulation of cell cycle-related genes such as *CDK2*, *CDCA5*, *CDC7*, *WEE1*, *E2F8*, *CCNA2*, *CDC6*, *CDC25A* and *CDC25C*.

Furthermore, the relation of the top 10 GO terms (all underexpressed) and significant DEGs identified various gene sets with crucial roles in DNA replication processes such as mini chromosome maintenance (*MCM*), centromer (*CEN*), cell division cycle (*CDC*), kinesin family (*KIF*), GINS complex subunit (*GINS*). Several top GO terms contain significantly modulated genes related to the minichromosome maintenance complex (*MCM*). MCM proteins are DNA helicases regulating and initiating DNA replication processes by unwinding duplex DNA, thus playing a pivotal role in cell division [82]. Various *MCMs* have been shown to be involved in tumorigenesis. *MCM3* for example, has been identified being overexpressed in several cancers including leukemia and lymphoma [83]. High *MCM2* expression was also suggested as a negative prognostic marker in DLBCL [84]. Down-regulation of *MCM5* by JQ1 BET inhibitor was already identified in thyroid cancer cells [85]. However, AZD as single agent induced only slight downregulation of *MCM* genes in our study. Further, the cell division cycle gene *CDC7* is highly expressed in a variety of tumors, suggesting a role as a biomarker [86,87]. Thus, targeted CDC7 inhibition is already under preclinical and clinical investigation (reviewed in [88]). *CDC7* and *MCM2* have been also suggested as prognostic markers in DLBCL [89].

*GINS1* and *GINS2* are likewise involved in DNA replication initiation and are upregulated in a variety of tumors, indicating potential as a prognostic marker [90,91]. CDC45, likewise downregulated by the combination, is a member of the CMG (CDC45/MCM2-7/GINS) complex. This complex has a pivotal role in DNA replication initiation by unwinding the DNA in order to initiate DNA synthesis [92,93]. The CMG complex is overexpressed in several cancers and is thus suggested as a prognostic marker and target for anticancer therapy strategies (reviewed in [94]). The significant downregulation of CMG complex members intimates its role in the underlying mechanism induced by Ento+AZD combined exposure.

Several of the identified genes related to the top 10 GO terms are involved in DNA replication processes. We further identified their functions in tumorigenesis or high expression in cancers, suggesting biomarker and prognostic marker potential.

### 4.7. Main GO Clusters in SU-DHL-4 after Combined Exposure

Furthermore, GO terms were condensed using REVIGO, summarizing the GO terms based on semantic similarities and they were then visualized using CirGO, which highlighted changes in GO clusters. In particular, the arising GO clusters enriched in the underexpressed DEGs revealed that more than 50% of identified GO terms are related to DNA replication and cell cycle processes, as with the top GO terms when simply sorted by combined score. The emerging GO clusters match with the biological data of the cell cycle blockade as well as the mode of action of BET inhibition by AZD and its interference in histone recognition.

The intervention in the biological processes is suggested to be mainly caused by the BET inhibition process, due to its major effects on biological and gene expression levels. However, the Ento addition leads to the specific gene and pathway deregulation caused by the combined exposure.

The combination-specific emerging GO terms as well as gene modulation are, for the most part, exclusive and therefore they provide an indication for a combination-specific gene set modulation by Entospletinib and AZD5153 combined exposure. Both the enhanced biological response as well as the strongly enhanced gene modulation by the combination suggest an advantageous intervention for DLBCL subtypes, which requires further investigation.

## 5. Conclusions and Future Perspective

Here, we identified a moderate synergy of simultaneous BET and SYK inhibition in a BL and DLBCL in vitro approach affecting cell cycle modulation but not apoptosis. Expression analysis characterized a distinct gene expression modulation, while the combination strongly increased this modulation. The combination-specific emerging DEGs and GO terms are mostly exclusive and therefore provide an indication for a combination-specific gene set modulation. Although the changes in the main processes are mainly provoked by AZD exposure, Ento addition leads to specifically modified gene and pathway deregulation. The main affected biological processes can be related to DNA replication and cell division.

The significantly differentially expressed genes *ADGRA2*, *MYB*, *TNFRSF11A*, *S100A10*, *PLEKHH3*, *DHRS2* and *FOXP1-AS1* presumably play a pivotal role of the Ento+AZD acting mode in SU-DHL-4 cells.

Future work remains to fully understand the underlying mechanism of the Ento+AZD combination. Prospectively, it remains to clarify which modulated genes are key regulators by including functional analyses and extending the work to other B-lymphoma cell lines. Proteomic analyses as well as STRING analyses could further validate the affected biological processes investigated by RNAseq. A full understanding of the Ento+AZD combination-induced effects will also need to involve 3D and in vivo models. This is especially important for a better transferability and investigation of clinical value.

## Data Availability

Data has been uploaded to GEO (NCBI): GSE207381, GSE207382, GSE207383.

## Supplementary Materials

The following supporting information can be downloaded at: https://www.mdpi.com/article/10.3390/cancers14194691/s1, Figure S1: IC50 calculation based on 72 h proliferation; Figure S2: Evaluation of cell viability after Entospletinib exposition; Figure S3: Enlarged figure of morphology after BET and SYK inhibition in SU-DHL-4 cell line; Figure S4: Enlarged figure of morphology after BET and SYK inhibition in DG-75 cell line; Figure S5: Cell cycle analysis after BET inhibition by flow cytometry; Figure S6: Bliss synergy based on cell cycle distribution; Figure S7: Gene expression modulation in SU-DHL-4 identified by Microarray Analyses; Figure S8: Gene expression modulation in DG-75 identified by Microarray Analyses; Figure S9: Gene intersections between cell lines and platforms of combined Ento+AZD exposure; Figure S10: Gene intersections between all treatment conditions in SU-DHL-4 by microarray analyses; Figure S11: Gene intersections between all treatment conditions in DG-75 by microarray analyses; Figure S12: Differential expression of identified genes in canine primary B-cell lymphoma samples compared to canine non-neoplastic samples; Figure S13: Ensuing Gene Ontology terms by Ento+AZD combination and single agents Ento or AZD in SU-DHL-4 cells; Figure S14: Chord plots of the top10 GO terms by Ento and AZD single agents and Ento+AZD combined exposure in SU-DHL-4; Figure S15: GO term intersections between treatment conditions in SU-DHL-4 by RNASeq; Table S1: RNASeq DEGs; Table S2: SU-DHL-4 Microarray; Table S3: DG-75 Microarray; Table S4: platform comparison; Table S5: RNAseq Gene Intersection; Table S6: GO terms; Table S7: GO + DEG; Table S8: GO intersections; Table S9: GO significant Overlaps.
